# Trends for Percutaneous Tracheostomy in Italian Acute Care Setting over a 5-Year Period

**DOI:** 10.3390/medicina59081444

**Published:** 2023-08-09

**Authors:** Maria Vargas, Pasquale Buonanno, Stefania De Simone, Gennaro Russo, Carmine Iacovazzo, Giuseppe Servillo

**Affiliations:** 1 Department of Neurosciences, Reproductive and Odontostomatological Sciences, University of Naples “Federico II”, 80138 Naples, Italy; 2 Department of Political Sciences, University of Naples Federico II, 80138 Naples, Italy; 3 Otolaryngology Head and Neck Surgery Unit, “Azienda Ospedaliera di Rilievo Nazionale dei Colli, Ospedale Monaldi”, 80138 Naples, Italy

**Keywords:** percutaneous tracheostomy, costs, length of stay, risk factors

## Abstract

*Background*: Tracheostomy is a widespread procedure usually performed with a percutaneous approach for prolonged mechanical ventilation. Little is known about the population-based trends for percutaneous tracheostomies (PT). The aim of this study was to evaluate the order to analyze the characteristics, rates, and costs of PTs performed in Italy from 2009 to 2014. *Methods*: We analyzed 102,646 PTs performed in Italy between 2009 and 2014. We obtained the data of patients from the section of the discharge report of the Italian Ministry of Health (National Archive for Hospital Discharge Form, Ministry of Health) about age, gender, length of stay (LOS), hospital types, and hospital region for code 541 and 542 for the years 2009, 2010, 2011, 2012, 2013 and 2014. Our additional source of data was the Annual Discharge Reports of the Italian Ministry of Health. *Results*: In this study, including 102,646 PTs performed from 2009 to 2014, we found that (1) the rates of PTs significantly decreased over time; (2) PTs were mostly performed in patients aged less than 65 years and hospitalized in ICUs for less than 40 days; and (3) the costs of PTs severely decreased over time, with a breakpoint between 2011 and 2012. *Conclusions*: Percutaneous tracheostomy is still a procedure frequently performed in the setting of acute care. Although percutaneous tracheostomy still results in high medical care reimbursement, it is a safe and cost-saving procedure.

## 1. Introduction

Tracheostomy is a common procedure with a low complication rate. This procedure can be performed either by an intensive care unit (ICU) physician with a percutaneous technique in the patient’s bed or by a surgeon with a traditional open approach [1]. Tracheostomy is mainly performed in ICUs for patients with respiratory and neurologic conditions that require mechanical ventilation [2]. Indeed, general indications for percutaneous tracheostomy (PT) in critically ill patients include prolonged mechanical ventilation and difficultly weaning [2], although upper airway obstruction secondary to intrinsic or extrinsic laryngeal pathologies is the main indication [3,4]. Real-world data about tracheostomies are not available, since national or international registries about this procedure are not available [5]. It can thus be challenging to find national data on the number of patients managed with PTs [5], but, nevertheless, according to a survey-based study undertaken by the National Confidential Enquiry into Patient Outcome and Death (NCEPOD), the incidence of tracheostomy is up to 30% of acute hospital admissions, and nearly 70% of those were PT [6]. Mehta et al. reported 20-year population-based trends analyses evaluating the use of tracheostomy for prolonged mechanical ventilation [7]. Nonetheless, little is known about the trends for PTs performed in ICU.

On the other hand, risk factors associated with the receipt of PTs among hospitalized patients were extensively studied by the current literature, showing that younger patients with a severe acute illness were more likely to receive a tracheostomy [7].

In this context, we obtained individual patient data from Italian Ministry of Health in order to analyze the characteristics, rates, and costs of PTs performed in Italy from 2009 to 2014.

## 2. Methods

### 2.1. Study Design

We selected the diagnosis-related group (DRG) codes for the tracheostomy procedure from the Italian system of DRG version 24 [8]. Accordingly, we found that the code 541 (tracheostomy) was associated with 96 h of mechanical ventilation and major surgery, and the code 542 (tracheostomy) was associated with 96 h of mechanical ventilation without major surgery. We obtained individual patient data from the section of the discharge report of the Italian Ministry of Health (National Archive for Hospital Discharge Form, Ministry of Health) about age, gender, length of ICU stay (LOS), hospital types, and hospital region for code 541 and 542 for the years 2009, 2010, 2011, 2012, 2013, and 2014. We collected data about tracheostomy admissions, acute care admissions, acute care admissions in public and private hospitals, hospital length of stay, total duration of hospital stay, hospital length of stay, 1-day costs, and total costs from the Annual Discharge Reports of the Italian Ministry of Health, where we extracted data from 2009 to 2014 [9]. We also collected, from the Italian Institute of Statistics (ISTAT), the resident, pediatric, and adult populations for the study years.

### 2.2. Statistical Analysis

Data were reported as means and standard deviations (±SD) or proportions as appropriate. A linear regression was used to assess the trends of PTs over the years. Statistical significance (*p*) was set at 0.05. Statistical analysis was obtained with SPSS (version 20.0, IBM^®^, Armonk, NY, USA).

## 3. Results

In this study, we included 102,646 PTs performed from 2009 to 2014. Table 1 showed the characteristics of patients receiving tracheostomy. A total of 16,000 patients per year received a tracheostomy in the ICU. The rate of PTs significantly decreased over time (*p* = 6.6 × 10^−11^), while the rate of surgical tracheostomy did not change (Table S1 in Supplementary Materials).

Patients aged less than 65 years were more likely to receive PT over time (*p* = 1.38 × 10^−6^). The rate of PTs increased over time within the 40 days of ICU stay (*p* < 0.05) and slightly decreased after 40 days (*p* < 0.05). The rate of PTs over time decreased in the public hospital (*p* = 0.0221).

**Figure 1:**
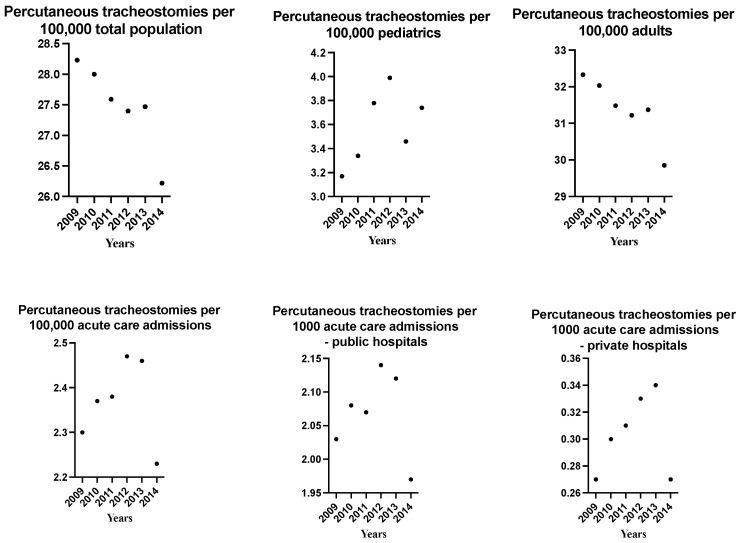
Rates of percutaneous tracheostomy over the years.

Figure 1 showed the incidence of PT over the years. The rates of PTs were statistically different over the years (*p* < 0.001). PTs for 100,000 pediatric patients peaked in 2012. PTs for 100,000 adult patients slightly decreased over time. PTs per 1000 acute care admissions peaked in 2012. Surgical tracheostomy remained stable over time in pediatric and adult patients and per 1000 ICU admission (see Table S2).

Table 2 showed the LOS of patients receiving PTs. PTs had a duration of hospital stay of 43 days in 2009 that decreased to 41 days in 2014 with statistical significance, even though the total duration of stay decreased over the considered years (*p* < 2 × 10^−16^).

**Figure 2:**
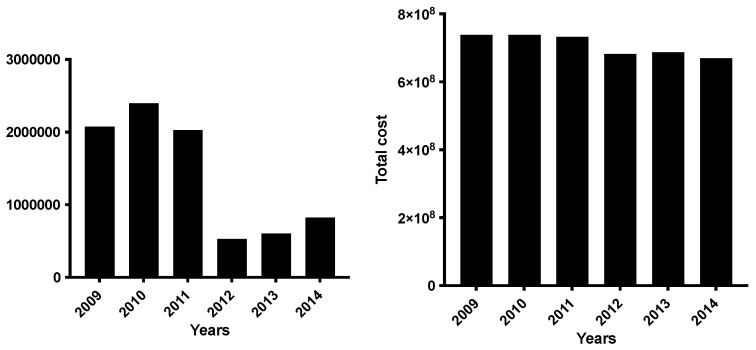
One-day and total-cost of patients receiving a percutaneous tracheostomy. Cost is expressed in EUR (€).

Figure 2 showed the 1-day and total costs of patients with PTs. The 1-day cost and the cost of PTs differed over the years with statistical significance (*p* < 0.001).

The 1-day and total costs significantly decreased over time, while the total cost of PTs had a peak in 2012

## 4. Discussion

In this study, including 102,646 PTs performed from 2009 to 2014, we found that (1) the rate of PTs significantly decreased over time; (2) PTs were mostly performed on patients aged less than 65 years and hospitalized in ICU for less than 40 days; and (3) the costs of PTs severely decreased over time, with a breakpoint between 2011 and 2012.

PT is a common procedure mainly performed for critically ill patients admitted in ICU [7,10], and when the percutaneous approach is contraindicated, surgical tracheostomy can be performed instead [4,10]. PT is one of the most frequent procedures performed in the ICU [7], and it may add potential benefit to the clinical management of critically ill patients by increasing patients’ comfort, reducing the need for sedation, facilitating the weaning process, and hastening ICU discharge [7].

Indications for tracheostomy in ICU included a wide range of options [7]. Tracheostomy is indicated in case of prolonged mechanical ventilation, difficult weaning, neurologic disease, incapability to protect the airways, and inability to manage secretions [11].

The trend analysis of PT is crucial because only few studies reported the real-world data about tracheostomies in critically ill patients. Accordingly, PTs have doubled during the last 20 years [7]. In our analysis, the rate of PTs has slightly decreased over a period of 6 consecutive years. On the other side, the rate of surgical tracheostomy did not change over time because this procedure still had a place in the management of acute critically ill patients (i.e., prolonged mechanical ventilation, difficult weaning). Surgical tracheostomy is a technique mainly performed for head and neck disease, and, in this case, its trend strictly depends on the epidemiology of these pathologies. However, surgical tracheostomy is a common procedure performed in ICUs in different countries [12].

The popularity of surgical tracheostomy in ICUs is due to the fact that it is easy and safe to perform with a fast learning curve [12]. Indeed, surgical tracheostomy is mainly performed in ICUs with more than eight beds [12]. This is probably due to the fact that the surgical technique involves less ICU staff and is mainly performed by the surgical staff [12]. Tracheostomy is a widely used alternative to translaryngeal endotracheal intubation in acute care settings. It can be easily performed both at the bedside and in the operating room; thus, its implementation is highly feasible [12]. It was surprising that between 2009 and 2014, the rate of PT decreased a little, but, nonetheless, the percentages of PTs per 1000 acute care admissions remained quite stable during the years.

The decrease in costs for PTs over time might have discouraged physicians from performing a standardized but risky procedure in critically ill patients. However, even the severity of the illness may be responsible for PT. On the other hand, factors driving the increase in tracheostomies in patients requiring acute care are unclear, and a number of processes may result in the high utilization of tracheostomies [13]. PTs may add several advantages in the management of acute patients in terms of patient mobility and respiratory physiology [14]. On the contrary, according to Fernandez et al. [15], tracheostomized critically ill patients had higher risks of death, the need of vasoactive drugs, and transfusion and acute renal failure than patients without tracheostomy, figuring out a more complex acute admission.

According to our analysis, PTs were mostly performed in patients aged less than 65 years and hospitalized in ICUs for less than 40 days. Age-specific incidence of respiratory failure was 99.9 cases per 100,000 for patients between 45 and 54 years of age, but this number suddenly increased to 493.5 cases per 100,000 for patients 65 to 74 years of age [16,17]. Probably, increasing age is associated with the prolonged MV, which is the most frequent indication for PTs in ICU [2]. Furthermore, procedures to reduce hospital and ICU LOS have risen over time [7]. Discharging a patient from ICU may be cost saving, and, in this view, PTs may fasten the hospital discharge of patients to long-term facilities [18].

Critical care costs have been rarely studied, even if there is a high utilization resource use [19]. Recently, the American Thoracic Society recommended a decision in order to keep the quality of care but reduce costs [20]. Tracheostomy costs have been previously analyzed by Mehta et al. and Cox et al., resulting in high medical care reimbursement [7,21]. In our study, we found a high reimbursement for PTs, even though the trends decreased over the years, with a breakpoint between 2011 and 2012. This breakpoint probably referred to a reduction in medical care spending implemented in those years. Nevertheless, PTs belong to the major diagnostic category with the highest reimbursement in DRG codes. Patients requiring a tracheostomy comprise only a small portion of hospitalizations, but they accounted for disproportionately high costs [21]. In our hospital system, about 17,000 PTs were responsible for almost 700 million EUR in costs.

## 5. Conclusions

In this patient data analysis, including 102,646 tracheostomized patients, we found that percutaneous tracheostomy is still a procedure frequently performed in ICU, and its rate per 1000 acute care admissions remained quite stable during the years.

Furthermore, the recent pandemic has seen a dramatic increase in patients requiring mechanical ventilation and higher rates of tracheostomy in patients with COVID-19, and, according to this, tracheostomy still has an important implication for the management of patients in ICUs.

## 6. Limitations

This study has some limitations that need to be addressed. First, we were able to collect only data from 2009 to 2014 because the codes 482, 541, and 542 for tracheostomies included in the Italian system of DRG version 24 had been used since 1 January 2009. Second, the code 542—tracheostomy associated with 96 h of mechanical ventilation without major surgery—also included cases of extra corporeal membrane oxygenation (ECMO). However, the Italian ECMO cases were very rare. According to the Italian ECMO network, 5 patients received ECMO between 2009 and 2010, 67 patients between 2011 and 2012, and 17 patients between 2011 and 2012. Third, we did not have data about the reason for ICU admissions. Fourth, we did not have data about tracheostomies in COVID-19 patients, since the procedure to have data from the Ministry of Health is no longer available. According to this, the data referred to an older period of time.

## Figures and Tables

**Table 1:** Characteristics of patients receiving tracheotomies.

Year	2009	2010	2011	2012	2013	2014	*p*
Percutaneous Tracheostomies (n)	16,953	16,893	16,726	16,274	16,394	15,938	6.6 × 10^−11^
Age,							1.38 × 10^−6^
Mean (SD)	65 (24)	65 (18)	64 (19)	64 (18)	64 (17)	64 (17)	
≤65 yr %	79.87	79.77	79.71	79.17	78.82	78.59	
66–84 yr %	17.60	17.59	17.53	17.95	18.18	18.35	
≥85 yr %	2.53	2.64	2.76	2.88	3.00	3.06	
Gender %							
M	62.3	62.0	62.6	62.6	62.6	63.2	0.0544
F	37.7	38.0	37.4	37.4	37.4	36.8	
Length of ICU stay							
≤20 ds %	25.3	25.0	25.5	26.7	26.9	26.6	5.52 × 10^−5^
21–39 ds %	32.8	32.5	32.7	33.6	33.6	33.7	0.0161
≥40 ds %	41.9	42.5	41.8	39.7	39.5	39.7	7.56 × 10^−8^
Hospital type,							
Public %	88.11	88.31	87.54	87.21	86.62	86.21	0.0221
Private with reimbursement %	11.84	11.67	12.44	12.77	13.35	13.73	1.03 × 10^−10^
Private without reimbursement %	0.05	0.02	0.02	0.012	0.03	0.06	0.705
Hospital region,							
North %	50.8	49.2	49.4	48.7	46.6	46.7	2.22 × 10^−10^
Mid %	21.3	22.5	22.4	22.7	23.1	23.1	0.000313
South %	27.9	28.3	28.2	28.6	30.3	30.2	4.47 × 10^−7^

**Table 2:** Length of ICU stay (LOS) of patients receiving percutaneous tracheostomy. * Adjusted for age, gender, hospital type and region.

Year	2009	2010	2011	2012	2013	2014	*p*
LOS, mean (SD)	43 (35)	43 (36)	43 (40)	42 (38)	42 (39)	41 (36)	<2 × 10^−16^ *
Total duration of stay, days	724,713	732,025	727,954	684,119	684,226	661,277	<2 × 10^−16^

## Data Availability

Data are available from the corresponding author after motivated request.

## Supplementary Materials

The following supporting information can be downloaded at: https://www.mdpi.com/article/10.3390/medicina59081444/s1, Table S1: characteristics of patients receiving tracheostomies. PT: percutaneous tracheostomy, ST: surgical tracheostomy. SD: standard deviation; yr: years; M: male; F: female; ds: days; Table S2: the rates of percutaneous tracheostomy over the years. PT: percutaneous tracheostomy, ST: surgical tracheostomy.
