# Supplementary material for: Trends for Percutaneous Tracheostomy in Italian Acute Care Setting over a 5-Year Period
Source: Medicina (Kaunas). 2023 Aug 9;59(8):1444. doi: 10.3390/medicina59081444 (PMC10456237; doi:10.3390/medicina59081444)
Supplement: Supplementary file 1 [file medicina-59-01444-s001.zip › medicina-2504897-supplementary.pdf]

Supplementary

Table S1. characteristics of patients receiving tracheostomies. PT: percutaneous tracheostomy, ST: surgical tracheostomy. SD: standard deviation; yr: years; M: male; F: female; ds: days.

	**2009**		**2010**		**2011**		2012		2013		2014	
	**PT**	**ST**	**PT**	**ST**	**PT**	ST	PT	ST	PT	ST	PT	ST
Tracheostomies (n)	16,953	4763	17211	4505	16998	4718	17012	4704	17164	4552	17318	4398
Age,												
mean (SD)	65(24)	64(19)	65(18)	64(13)	64 (19)	64(12)	64(17)	64(13)	64 (17)	64(13)	65(17)	65(13)
≤ 65 yr %	31	11.3	31.6	10.8	33.8	11	34.7	9.8	31.5	11	30.9	10.2
66-84 yr%	43	9.8	42.5	9.2	40.4	10	38.8	10.6	40.3	9.1	38.9	9.2
≥ 85 yr %	4.1	0.8	3.8	0.8	3.8	0.8	3.5	1	3.7	0.9	3.7	0.8
Gender %, M	48.6	17.2	48.3	15.9	48.8	17.2	46.9	16.7	47.2	16.1	46.4	15.3
F	29.4	4.7	29.7	4.6	29.2	4.8	28	5	28.3	4.9	27	5
Length of stay,												
≤ 20 ds %	19.8	10.9	19.5	10.6	19.9	11.3	20	11.4	20.3	11.4	19.5	10.9
21-39 ds %	26.8	8	26.4	7.2	26.8	7.6	26.3	7.6	26.3	6.9	25.8	6.9
≥ 40 ds %	31.5	3.1	32.2	2.8	31.3	3.1	28.7	2.7	28.9	2.6	28.1	2.4
Hospital type, Public %	68.7	19.6	68.8	18.2	68.3	19.3	55.4	18.8	55.4	18.3	63.3	17.6
Private with reimbursement %	9.2	2.3	9.1	2.3	9.7	2.5	9.6	2.8	10.1	2.5	10	2.7
Private without reimbursement %	0	0	0	0	0	0.1	0	0	0	0	0	0
Hospital region, North %	45.6	15.2	44.4	14	45.1	14.8	43.2	14.3	41.9	13.8	41	13.2
Mid %	13.1	3.8	13.6	3	13.4	3.2	12.6	3.1	13.5	2.8	12.5	2.7
South %	19.4	3	29.7	3.7	19.6	4.1	19.2	4.3	20.1	4.3	19.9	4.3

Table S2. The rates of percutaneous tracheostomy over the years. PT: percutaneous tracheostomy, ST: surgical tracheostomy.

	**2009**		**2010**		**2011**		2012		2013		2014	
	**PT**	**ST**	**PT**	**ST**	**PT**	ST	PT	ST	PT	ST	PT	ST
Tracheostomies per 100,000 total population	28.2	7.9	28.5	7.5	28.6	7.9	28.6	7.9	28.7	7.6	28.8	7.2
Tracheostomies per 100,000 pediatrics	3.7	0.2	3.7	0.15	4.2	0.15	4	0.1	3.7	0.15	3.6	0.15
Tracheostomies per 100,000 adults	33.2	9.5	33.6	8.9	32.8	9.3	33	9.3	33.8	9.1	33.5	8.6
Tracheostomies per 1,000 acute care admissions	2.3	0.6	2.3	0.6	2.4	0.65	2.5	0.7	2.6	0.7	2.7	0.7
Tracheostomies per 1,000 acute care admissions – public hospital	2.6	0.7	2.6	0.7	2.7	0.7	2.7	0.7	2.8	0.7	2.7	0.7
Tracheostomies per 1,000 acute care admissions – private hospital	1.1	0.3	1.2	0.3	1.1	0.3	1.2	0.4	1.4	0.3	1.5	0.4
